# *De novo* Mutations From Whole Exome Sequencing in Neurodevelopmental and Psychiatric Disorders: From Discovery to Application

**DOI:** 10.3389/fgene.2019.00258

**Published:** 2019-04-03

**Authors:** Weidi Wang, Roser Corominas, Guan Ning Lin

**Affiliations:** ^1^Shanghai Mental Health Center, School of Biomedical Engineering, Shanghai Jiao Tong University School of Medicine, Shanghai, China; ^2^Shanghai Key Laboratory of Psychotic Disorders, Shanghai Mental Health Center, Shanghai, China; ^3^Brain Science and Technology Research Center, Shanghai Jiao Tong University, Shanghai, China; ^4^Departament de Genètica, Microbiologia i Estadística, Facultat de Biologia, Universitat de Barcelona, Barcelona, Spain; ^5^Centro de Investigación Biomédica en Red de Enfermedades Raras, Valencia, Spain; ^6^Institut de Biomedicina de la Universitat de Barcelona, Barcelona, Spain; ^7^Institut de Recerca Sant Joan de Déu, Esplugues de Llobregat, Barcelona, Spain

**Keywords:** whole exome sequencing, neurodevelopmental and psychiatric disorder, *de novo* mutation, network analysis, clinical implementation

## Abstract

Neurodevelopmental and psychiatric disorders are a highly disabling and heterogeneous group of developmental and mental disorders, resulting from complex interactions of genetic and environmental risk factors. The nature of multifactorial traits and the presence of comorbidity and polygenicity in these disorders present challenges in both disease risk identification and clinical diagnoses. The genetic component has been firmly established, but the identification of all the causative variants remains elusive. The development of next-generation sequencing, especially whole exome sequencing (WES), has greatly enriched our knowledge of the precise genetic alterations of human diseases, including brain-related disorders. In particular, the extensive usage of WES in research studies has uncovered the important contribution of *de novo* mutations (DNMs) to these disorders. Trio and quad familial WES are a particularly useful approach to discover DNMs. Here, we review the major WES studies in neurodevelopmental and psychiatric disorders and summarize how genes hit by discovered DNMs are shared among different disorders. Next, we discuss different integrative approaches utilized to interrogate DNMs and to identify biological pathways that may disrupt brain development and shed light on our understanding of the genetic architecture underlying these disorders. Lastly, we discuss the current state of the transition from WES research to its routine clinical application. This review will assist researchers and clinicians in the interpretation of variants obtained from WES studies, and highlights the need to develop consensus analytical protocols and validated lists of genes appropriate for clinical laboratory analysis, in order to reach the growing demands.

## Introduction

The susceptibility to neurodevelopmental and psychiatric (NDP) disorders involves polygenic, multi-effect, and complex genetic structures. Large-scale twin and population-based studies were able to show that genetics accounts for 30–80% of disease liability for many neuropsychiatric disorders (Gandal et al., 2016). An early indication of the genetic involvement in major NDP disorders was their association with rare Mendelian disorders, each with distinctive morphologic, cognitive, and neuropsychiatric phenotypes, such as Fragile X, Angelman, or Rett syndromes (De Boulle et al., 1993; De Hert et al., 1996; Inoue and Lupski, 2003; Betancur, 2011; Blair et al., 2013). Due to the limited power and systematic confounders such as population stratification bias, the early linkage and candidate gene studies often yielded disperse findings (Kohler and Bickeboller, 2006; Price et al., 2010). In contrast, the unbiased large-scale genome-wide interrogation, such as genome-wide association studies (GWAS), of common genetic variation in large cohorts has become a popular detection methodology and study design to identify risk factors in neuropsychiatric diseases, such as autism spectrum disorders (ASD), schizophrenia (SCZ), intellectual disability, and/or developmental delay (ID/DD), obsessive-compulsive disorder (OCD) or bipolar disorder (BD) and has yielded more robust results (Epi4k Consortium et al., 2013; Schizophrenia Working Group of the Psychiatric Genomics Consortium et al., 2014; Hou et al., 2016; Liu et al., 2016a; The Autism Spectrum Disorders Working Group of The Psychiatric Genomics Consortium, 2017; Trampush et al., 2017; Ikeda et al., 2018; Pasman et al., 2018). Interesting GWAS discoveries have been made on a wide variety of neuropsychiatric disorders and clinical traits, many of which have been covered in other in-depth reviews (Sullivan, 2010; Visscher et al., 2012, 2017; Collins and Sullivan, 2013; Horwitz et al., 2018). Therefore, the scope of our review is mainly focused on the usage of next generation sequencing (NGS) technology, specifically whole exome sequencing (WES), as an additional approach in neuropsychiatric disorder studies, in an attempt to uncover the missing genetic basis of the disease etiology. There are two commonly used study strategies for discovering disease-associated variants using WES. One is the exome case-control study to find the rare inherited variants, which usually require a large sample size to identify the significant variants, such as in SCZ (Genovese et al., 2016), BD (Goes et al., 2016), and OCD (McGrath et al., 2014). A comprehensive review has been done by Gratten et al. (2014) on exome case-control studies and has contributed greatly to our understanding of neuropsychiatric diseases. The other one, is the *de novo* variants (DNV) discovery, based on trio/quad studies with relatively smaller sample sizes as an alternative strategy with great success in disorders, such as ASD (De Rubeis et al., 2014; Iossifov et al., 2014; O'Roak et al., 2014) and ID/DD (Gilissen et al., 2014; Short et al., 2018). Thus, in this review, we focus on the DNV studies, their significance and clinical translations. Here, we summarize the recent repertoire of *de novo* events detected from WES family-based trio (two unaffected biological parents and the affected child) or quad (two unaffected biological parents, the affected child, and one unaffected sibling) studies in NDP disorders and provide a detailed overview of some of the most successful integrative methods for DNM analyses. Finally, we also review the current state of WES application in NDP disorder clinical diagnosis.

## Dissecting Neurodevelopmental and Psychiatric Disorders by Identifying *de novo* Rare Genetic Variants

### Usage of Whole Exome Sequencing in NDP Disorders to Detect DNMs

**Figure 1:**
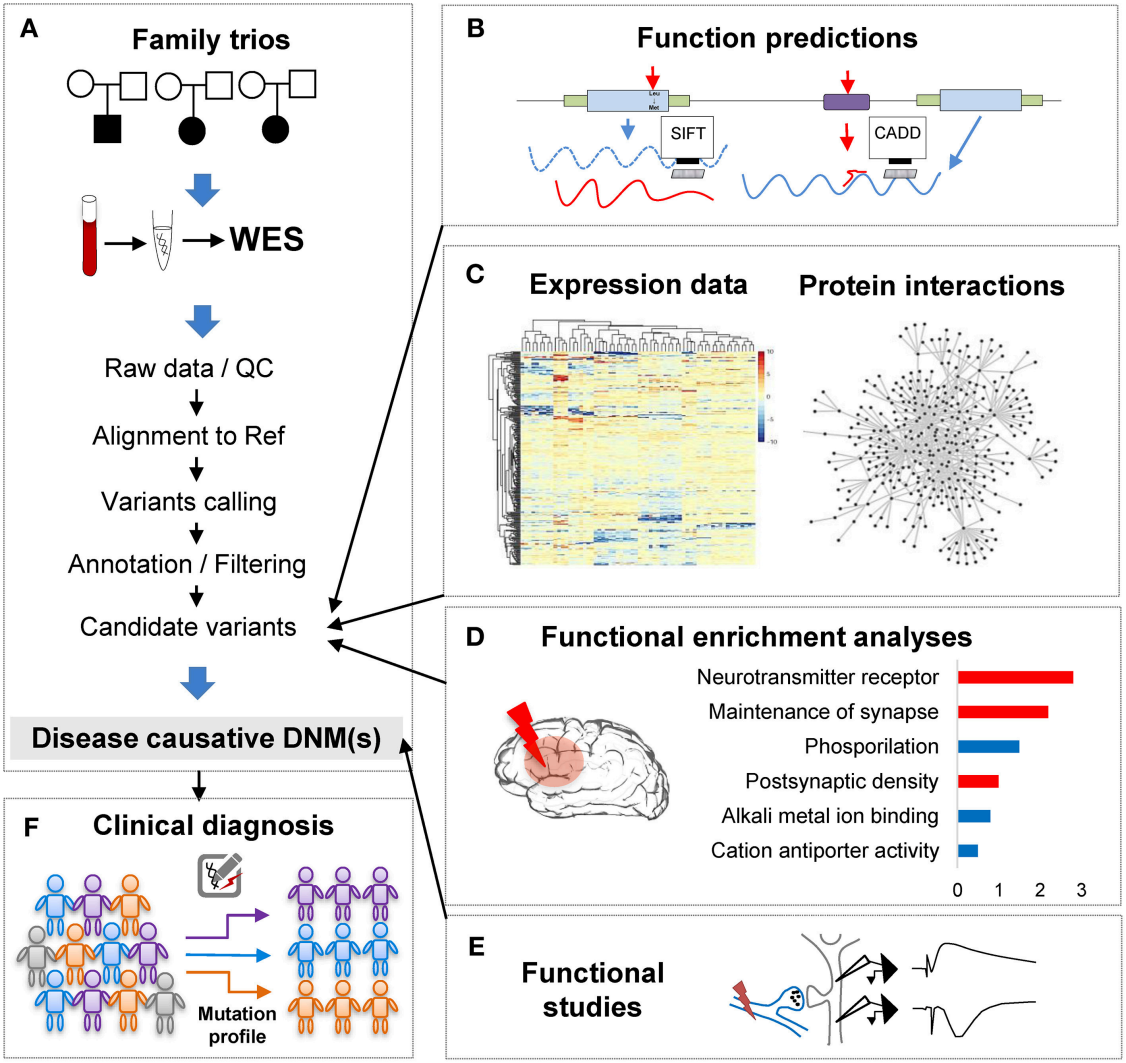
The analytical pipeline for DNMs detected from exome studies in psychiatric disorders. **(A)** The discovery flow of DNMs from trios, including sample collection, data Quality Control (QC), alignment, variants calling, annotation. **(B)** The functional annotation step, e.g., applying computational tools, such as SIFT and CADD to predict the functional consequences of the detected mutation. **(C)** The large-scale data integration step to investigate the underlying disease genetics. **(D)** The functional enrichment analyses and interpretation step, which help understand the disease etiology. **(E)** The functional studies phase is the experimental validation step. **(F)** The clinical application step of the DNM pipeline, which can utilize the verified DNMs as the mutation screening profile for clinical diagnosis in psychiatric patients.

Over the past decade, NGS has become increasingly popular for estimating the genetic etiology of Mendelian, complex, and undiagnosed disorders due to its scale and comprehensiveness (Bamshad et al., 2011; Goldstein et al., 2013; MacArthur et al., 2014; Zhu et al., 2014). In WES, the ~1.5% of the genome, encoding for proteins, is captured and then sequenced at lower costs and increases the interpretability of the identified variants, in contrast to the much more expensive whole genome sequencing (WGS) that surveys the entire genome space. To facilitate interpretation and decrease the cost of the whole genome sequencing (WGS), the portion of the genome related to protein coding regions is captured and sequenced in WES. Notably, studies of NDP disorders using WES and WGS have indicated that *de novo* mutations (DNMs) detected from trio- or quad-based familiar studies have important roles (Table 1), despite the genetic heterogeneity of types of disorders (Sullivan et al., 2012a; Gratten et al., 2013). Filtering DNV in a proband against unaffected parents, facilitates the interpretation of potential *de novo* pathogenic variants among all the detected ones (Figure 1A). For example, Neale and colleagues (Neale et al., 2012) performed one of the four first large WES cohort studies using a trio-based design to investigate the contribution of DNMs to ASD (Iossifov et al., 2012; Neale et al., 2012; O'Roak et al., 2012b; Sanders et al., 2012), which showcased the important role of DNMs in the pathogenesis of ASD. More importantly, Neale and colleagues also proposed a statistical framework to analyze whether individual genes carry significantly more DNMs than expected by chance. Furthermore, Sanders et al. estimated that penetrant DNMs in genes contribute to autism risk in ~11% of parent-child trio families (Sanders et al., 2015).

**Table 1:** Summary of published studies of DNMs found in large cohorts of patients with psychiatric disorders.

**Study**	Clinical phenotype^*^	**Number of individuals**	**Probands/controls**	**Sequencing method**
Vissers et al., 2010	MR	10	proband	WES
O'Roak et al., 2011	ASD	20	proband	WES
O'Roak et al., 2011	–	20	control	WES
Girard et al., 2011	SCZ	14	proband	Targeted
Xu et al., 2011	SCZ	53	proband	Targeted
Xu et al., 2011	–	22	control	Targeted
Sanders et al., 2012	ASD	238	proband	WES
Sanders et al., 2012	–	200	control	WES
O'Roak et al., 2012b	ASD	189	proband	WES
O'Roak et al., 2012b	–	31	control	WES
Neale et al., 2012	ASD	175	proband	WES
Iossifov et al., 2012	ASD	343	proband	WES
Iossifov et al., 2012	–	343	control	WES
Kong et al., 2012	ASD or SCZ	65	proband	WGS
Rauch et al., 2012	ID	51	proband	WES
Rauch et al., 2012	–	20	control	WES
de Ligt et al., 2012	ID	100	proband	WES
Xu et al., 2012	SCZ	231	proband	WES
Xu et al., 2012	–	34	control	WES
Barcia et al., 2012	EE	12	proband	WES
O'Roak et al., 2012a	ASD	2446	proband	Targeted
Michaelson et al., 2012	ASD	10	proband	WGS
Veeramah et al., 2013	EE	10	proband	WES
Jiang et al., 2013	ASD	32	proband	WGS
Gulsuner et al., 2013	SCZ	105	proband	WES
Gulsuner et al., 2013	–	84	control	WES
Epi4k Consortium et al., 2013	EE	264	proband	WES
Fromer et al., 2014	SCZ	623	proband	WES
McCarthy et al., 2014	SCZ	57	proband	WES
Takata et al., 2014	SCZ	231	proband	WES
Gilissen et al., 2014	ID	50	proband	WGS
Francioli et al., 2014	–	250	control	WES
Appenzeller et al., 2014	EE	356	proband	WGS
Hamdan et al., 2014	ID	41	proband	WES
De Rubeis et al., 2014	ASD	2303	proband	WES
Iossifov et al., 2014	ASD	2500	proband	WES
Iossifov et al., 2014	–	1911	control	WES
O'Roak et al., 2014	ASD	3486	proband	Targeted
O'Roak et al., 2014	ID	3486	proband	Targeted
O'Roak et al., 2014	–	2493	control	Targeted
Guipponi et al., 2014	SCZ	53	proband	WES
Yuen et al., 2015	ASD	170	proband	WGS
van Bon et al., 2016	ASD	7162	proband	Targeted
Krumm et al., 2015	ASD	2377	proband	WES
Krumm et al., 2015	–	1786	control	WES
Parker et al., 2015	ID	10	proband	WES
Kranz et al., 2015	SCZ	14	proband	WES
Kun-Rodrigues et al., 2015	Parkinson early-onset	21	proband	WES
Hashimoto et al., 2016	ASD	30	proband	WES
Turner et al., 2016	ASD	53	proband	WGS
Turner et al., 2016	–	43	control	WGS
Helbig et al., 2016	EE	1131	proband	WES
Cappi et al., 2016	OCD	20	proband	WES
Kataoka et al., 2016	BD	79	proband	WES
Tlemsani et al., 2016	ID	210	proband	Targeted
Halvardson et al., 2016	ID	39	proband	WES
Lelieveld et al., 2016	ID	820	proband	WES
Yuen et al., 2016	ASD	200	proband	WGS
Wang et al., 2016	ASD	1045	proband	Targeted
Deciphering Developmental Disorders Study et al., 2017	DD	4293	proband	WES
Stessman et al., 2017	ASD	4787	proband	Targeted
Stessman et al., 2017	ID	151	proband	Targeted
Stessman et al., 2017	DD	1133	proband	Targeted
Yuen et al., 2017	ASD	1740	proband	WGS
Kim et al., 2017	ADHD	11	proband	WES
Chen et al., 2017	ASD	116	proband	WES
Lanoiselée et al., 2017	Alzheimer early-onset	10	proband	Targeted
Willsey et al., 2017	Tourette Disorder	325	proband	WES
Nishi et al., 2017	SCZ	18	proband	WES
Hamdan et al., 2017	DEE	197	proband	WGS
Werling et al., 2018	ASD	519	proband	WGS

* *ADHD, Attention-Deficit/Hyperactivity Disorder; ASD, Autism Spectrum Disorder; BD, Bipolar Disorder; ID, Intellectual Disability; DD, Developmental Delay; DEE, Developmental and Epileptic Encephalopathies; EE, Epilepsy; MD, Mental Retardation; OCD, Obsessive-Compulsive Disorder; SCZ, Schizophrenia*.

### Summary of Published Exome DNMs in Neuropsychiatric Disorders

In the past few years, 66% of the large cohort studies that investigate DNMs of early onset psychiatric disorders published were carried out by WES, compared to only 18% by WGS and the remaining 16% by targeted sequencing, demonstrating the prominent role of the WES in mutation discovery (Table 1). These studies have identified, by the end of 2018 in denovo-db (Turner et al., 2017), more than 57,300 DNMs in 44,200 individuals with a variety of neuropsychiatric disorders estimating a rate of ~1.3 DNMs/individual (Table 1). Also, the decrease in cost of NGS over the years has resulted in an explosion of small WES studies from limited collections or index cases from all over the world (Smedemark-Margulies et al., 2016; Zhu et al., 2018). Manual curation and time are needed for all these variants to progressively be introduced in variant databases, such as 1000 Genomes (Gibbs et al., 2015), the National Heart Lung and Blood Institute's Exome Sequencing Project (ESP) (Exome Variant Server (http://evs.gs.washington.edu/EVS/), Database of Short Genetic Variations (dbSNP) (Sherry et al., 2001), expert-curated databases focused on variant information (locus-specific databases; LSDB) (Fokkema et al., 2011) or clinical information e.g., GeneReviews (http://www.ncbi.nlm.nih.gov/books/NBK1116/) and ClinVar (Landrum et al., 2016), where mutations are deposited by submitters, or collected by the private Human Mutation Database (HGMD) (Stenson et al., 2017). As for DNMs, a few databases are being developed hosting the collection of the DNMs across developmental and neuropsychiatric disorders and controls, such as denovo-db (Turner et al., 2017) and NPdenovo (Li et al., 2016). The same team also developed mirDNMR, a gene-centered database of background DNM rates in humans (Jiang et al., 2017). Still, these databases focus on the variant information, such as locations and frequencies. Therefore, a freely available unified systematic variance repository collecting the results from all the currently published variants fast, including medically relevant information, is needed to ensure the rapid translation of novel information to researchers and clinicians.

**Figure 2:**
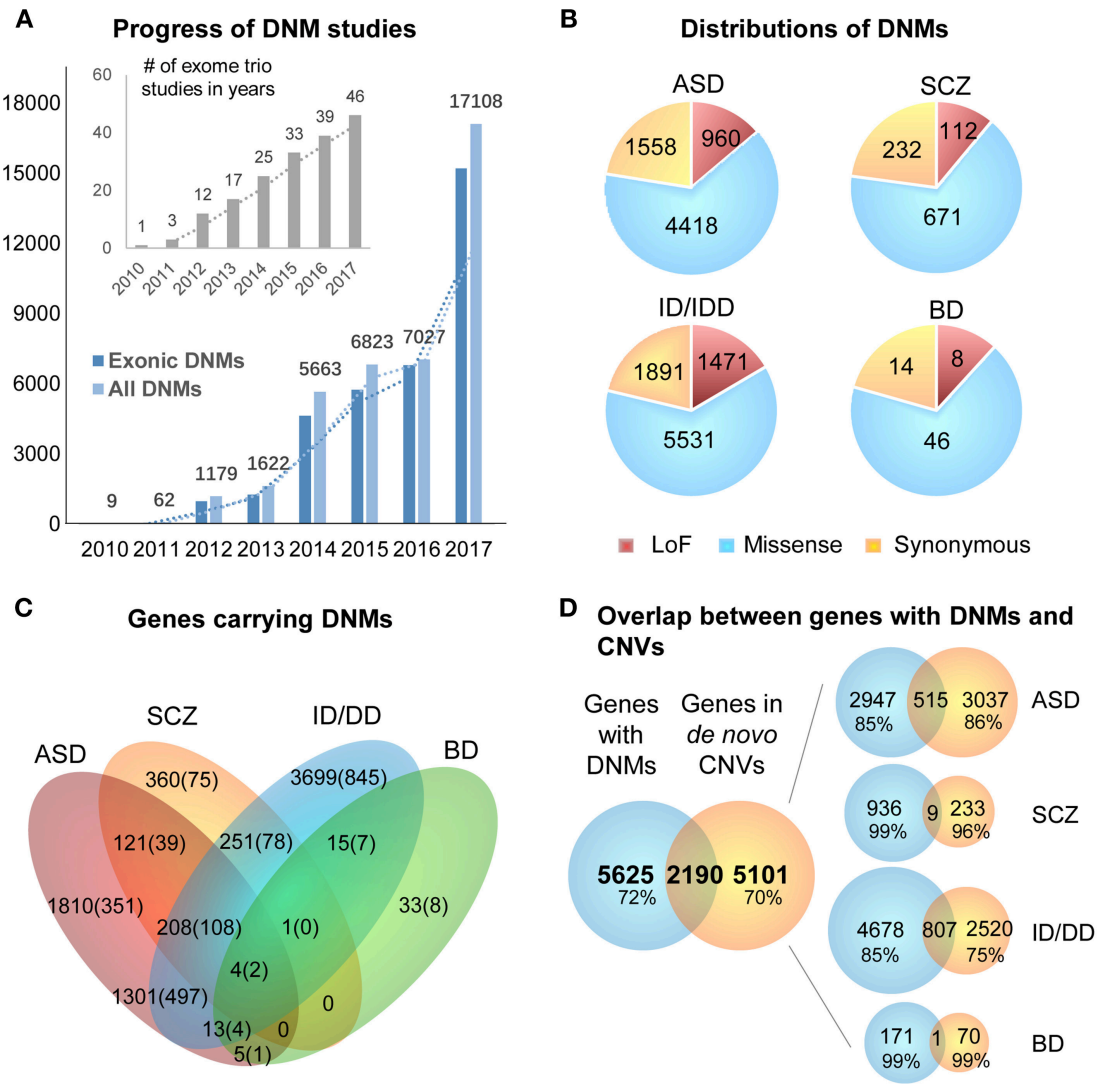
Summary of DNMs detected from exome studies in psychiatric disorders. **(A)** The overview of the numbers of WES studies in psychiatric disorders increased over the past few years (until the end of 2017). **(B)** The distribution of *de novo* LoF, missense, and synonymous mutations detected in four different disorders across large studies (Table 1). **(C)** Venn Diagram of the overlap of genes hit by DNMs from major studies of patients with psychiatric disorders. **(D)** The overlap between the genes carrying DNMs and genes hit by the *de novo* CNVs in four different disorders. ASD, Autism Spectrum Disorder; ID/DD, Intellectual Disability, Developmental Delay; SCZ, Schizophrenia; BD, Bipolar Disorder.

DNMs including single nucleotide variants (SNVs) and small insertions/deletions (indels) in exonic regions, are rare and generally considered to have a stronger disruptive effect on biological functions than inherited variants (Crow, 2000). Thus, DNMs provide a valuable insight into the genetic understanding and clinical interpretation of sporadic cases in which inheritance may be limited to explain disease etiology (Veltman and Brunner, 2012; MacArthur et al., 2014; Samocha et al., 2014). As a result, the number of WES studies and identified DNMs have increased rapidly over the past few years (Figure 2A). To facilitate the interpretation of DNMs from exonic and exon-flanking regions, they are usually categorized by their functional impacts (Figure 2B) as synonymous (23%) or non-synonymous variants (77%). As the former mutations typically have a silent effect, even some of them may contribute to alternative splicing and protein fold change (Sauna and Kimchi-Sarfaty, 2013), but their predictions are limited except biological experiments showing the results of these variants. Thus, we chose to focus on the non-synonymous DNMs. The latter is further classified into likely gene-damaging loss-of-function (LoF) variants (15%) (nonsense, frameshift indels, and splice-site mutations) and missense variants (62%). However, only a fraction of these DNMs is responsible for the clinical phenotypes. In an extensive study of >2,500 simplex families with ASD, 43% of LoFs, and 13% of the missense DNMs were estimated to be pathogenic (Iossifov et al., 2014). We observe that mutations from ASD and ID/DD contribute the most (86.6%) to the current repertoire of WES DNMs (Figure 2A), which is not surprising since 85% of the trio/quad samples presented these disorders and have been more systematically interrogated than others. Also, the distribution of variant types identified is similar across disorders (Figure 2B).

The excessive comorbidity between various neuropsychiatric diagnoses, such as ASD, ID/DD, SCZ, BD, OCD, Tourette syndrome, and ADHD makes the interpretation of the underlining disease etiology extremely difficult (Ronald et al., 2008; Lichtenstein et al., 2010; Rommelse et al., 2010; Faraone et al., 2012; Sullivan et al., 2012b; Chen et al., 2016; Hirschtritt et al., 2018; Liu and Wu, 2018; Shen et al., 2018). Another limiting factor is the large polygenicity of these diseases. Almost all NDP disorders are associated with potentially thousands of disease risk genes, each conferring variable effects. To this extent, accumulation of the genes carrying DNMs from WES in multiple NDP disorder studies provides an excellent opportunity for interrogating the underlying shared genetic component among various disorders. We used pLI scores (Lek et al., 2016) to prioritize and summarize the overlapped genes. The pLI score of a given gene indicates the probability that it belongs in the haploinsufficient category, wherein a single functional copy of a gene is insufficient to maintain its normal function and is extremely intolerant of LoF variation. Thus, we summarized the overlapped genes (Supplementary Table 1) with high pLI scores (pLI ≥ 0.9, extremely LoF intolerant) carrying DNMs between four different disorders, ASD, SCZ, ID/DD, and BP, in Figure 2C.

The disorders that shared the larger number of genes are ASD and ID/DD, but these observations could be explained by the extensive shared clinical phenotypes. These observations need to be considered with caution due to a large number of WES studies performed in these disorders. There are some noticeable genes carrying DNMs in patients from at least three different disorders, such as Chromodomain Helicase DNA Binding Protein 8 *CHD8* (ASD, ID/DD, SCZ), Lysine Methyltransferase 2C *KMT2C* (ASD, BP, ID/DD, SCZ), Chromodomain Helicase DNA Binding Protein 5 *CHD5* (ASD, ID/DD, SCZ), Sodium Voltage-Gated Channel Alpha Subunit 2 *SCN2A* (ASD, ID/DD, SCZ), Neurexin 1 *NRXN1* (ASD, ID/DD, SCZ), or Period Circadian Regulator 1 *PER1* (ASD, BP, ID/DD), suggesting that some biological processes are probably shared between neuropsychiatric disorders.

### Unique Contribution of *de novo* Events to the Understanding of Disease Etiology

A chromosomal structural variation (SV) is usually a rearrangement of a genomic region with variable size (50 bp−5 Mb) that can cause Mendelian disease and contribute to complex diseases (Stankiewicz and Lupski, 2010; Girirajan et al., 2011). It includes different types of alternations, such as inversions and balanced translocations or genomic imbalances (duplications and deletions), the latter commonly known as copy number variants (CNVs) (Weckselblatt and Rudd, 2015). The advent of chromosomal microarrays enabled the detection of large genomic *de novo* structural variants such as recurrent *de novo* (or inherited) CNV in trio studies (e.g., 1q21.1, 16p11.2, or 22q11.21) or ultra-rare or unique *de novo* CNVs. These discoveries provided an additional aspect of disease etiology and brought rare variants with large effect sizes to the forefront (Malhotra and Sebat, 2012). Especially with the advent of the high sequencing coverage, CNV calling from WES/WGS became more reliable (Trost et al., 2018), and a machine-learning algorithm based software (SV2) improved performance of the SV detection, including CNV, for genotyping deletions and duplications from paired-end sequencing data (Antaki et al., 2018; Brandler et al., 2018). It has been estimated that CNVs are responsible for a considerable percentage of the genetic causes in some psychiatric disorders such as ~10% of simplex cases of ASD (Sebat et al., 2007). However, *de novo* CNVs discovered in NDP disorders pose many challenges for interpretations, such as pleiotropy, incomplete penetrance, and difficulty to directly identify the pathogenic genes due to the fact that the affected region may contain no known gene (potentially affecting regulatory elements) or, on the contrary, a large and diverse set of genes. When overlapping the genes carrying DNMs from WES to the genes hit by *de novo* CNVs from arrays of four different disorders (ASD, BD, ID/DD, and SCZ), only <30% of them are shared (Figure 2D). These issues emphasize the need for different detection methods for interrogating the differential impact of molecular pathologies by different types of disease mutations. In consequence, WGS that covers the entire genome in trio or quad family-based studies is more comprehensive and capable of detecting a complete set of SNPs, SNVs, indels, and CNVs of an individual at the same time. Also, it could be a choice to replace the usual strategy of using multiple sequencing technologies to investigate all the variants in neuropsychiatric disorders.

## Interpreting rare genetic variants from exome studies

With the abundance of the genomic variants obtained from WES and WGS studies, one of the most significant challenges is to systematically interrogate the functional impact of the detected variants and identify the underlying affected pathway. Though the ultimate proof of DNMs contribution to the phenotypes is functional assays (Figure 1E), these tests are generally difficult to implement in such large scales systematically. To overcome this limitation, computational methods have been developed to investigate DNMs consequences and involve typically multiple steps: evaluation of the DNMs potential pathogenicity; incorporation of other variants such as CNVs or inherited DNMs; and finally, integrative analyses of data from other sources of evidence to enhance the understanding of the disease functional pathways (Figures 1B–D).

### Prediction of Functional Consequences of DNMs

One of the most important steps when the variants are obtained, is to functionally annotate them to distinguish deleterious variants from a considerable number of variants from the neutral background. Numerous efforts have been carried out to develop computational tools to functionally interpret both coding and non-coding genomic elements and to estimate the variants pathogenicity, such as SIFT (Ng and Henikoff, 2003), PolyPhen-2 (Adzhubei et al., 2010), GERP (Davydov et al., 2010), or CADD (Kircher et al., 2014) (Figure 1B). Pathogenicity evaluation can be a challenging task as the estimated results of different methods sometimes lack of the consistency, and functional assays are not systematically performed. To this end, several studies (Gnad et al., 2013; Dong et al., 2015; Miosge et al., 2015; Ionita-Laza et al., 2016) have extensively reviewed these computational annotation tools. Some of these studies divide the multiple tools according to the variant types predictions (Richards et al., 2015), while others compared non-coding genome pathogenicity scores using calculation methods based on machine learning approaches (Telenti et al., 2018). These summaries often include widely used tools such as SIFT (Ng and Henikoff, 2003), PolyPhen-2 (Adzhubei et al., 2010), or MutationTaster2 (Schwarz et al., 2014), which are based on the evolutionary conservation, which predicts the impacts by determining the conservation of an amino acid across species or based on protein 3D structures features, or both. Li et al. conducted a comprehensive evaluation of 23 methods for annotating missense variants using three independent benchmark datasets with 12 different performance measures (Li et al., 2018) and indicated that ReVe, a combination of REVEL (Ioannidis et al., 2016) and VEST3 (Carter et al., 2013a), had the best performance in prediction. However, comparative results should be often interpreted casually since the evaluation of these tools can be hindered by the problem of circularity (Grimm et al., 2015), such as different variants from the same protein occurring both in the datasets used for training and for evaluation. Nevertheless, these predictors are limited to estimate the impacts of SNVs on coding regions. Other computational tools, such as CADD (Kircher et al., 2014), LINSIGHT (Huang et al., 2017), FATHMM (Shihab et al., 2015), etc. have shown certain but limited power in predicting consequences of non-coding variants. Some have developed integrative tools that incorporate several of these algorithms. For example, dnNSFP (Liu et al., 2016b) integrated various computation tools such as SIFT (Ng and Henikoff, 2003), PolyPhen-2 (Adzhubei et al., 2010), and CADD (Kircher et al., 2014), and was developed to be a one-stop database for variant functional predictions and annotations. ANNOVAR (Wang et al., 2010) is another comprehensive annotation tool, which incorporates functional deleteriousness prediction scores from the dbNSFP, variants reported in the ClinVar database, variants reported in dbSNP, etc. These tools intend to interpret SNVs and indels through evaluating their functional impacts on genes, reporting all functional relevance scores from different computational tools, assessing conservation levels of the impacted region, and interrogating the position variability frequency in databases such as 1000 Genomes Project (Gibbs et al., 2015), dbSNP (Sherry et al., 2001), and ExAC (Lek et al., 2016). Since their development, both dnNSFP and ANNOVAR have become popular and widely used variants annotation applications.

Furthermore, applying neural networks (LeCun et al., 2015; Salakhutdinov, 2015) in annotating genomics information by sequences has become prevalent in recent years and indicates the start of the deep learning era for computational biology (Jones et al., 2017). Several WES studies have reported some small sets of DNMs hitting the non-coding regions of the genes along with exonic DNMs (Supplementary Table 2), which would require additional interpretations. Two recent methods, DeepSEA (Zhou and Troyanskaya, 2015) and DeepBind (Alipanahi et al., 2015), are great examples of applying the deep learning to model the sequence specificity in various level, particularly on variants hitting at non-coding regions. DeepSEA reached at the single nucleotide resolution to predict transcription factor binding and DNAse sensitivity. DeepBind can forecast the sequence specificity of DNA/RNA- protein binding. Their performance is found to be better than any existing conventional method for predicting non-coding variants consequences. DeepSEA predicts chromatin effects of sequence variations with single-nucleotide sensitivity, by directly learning a regulatory sequence code from large-scale chromatin-profiling data, including transcription factors binding, DNase I sensitivity and histone-mark profiles. DeepBind interrogates the sequence variants by integrating experimental data with a deep convolutional neural network to indicate how variations affect binding within a specific sequence. Compared to DeepSEA, DeepBind analyzes the binding affinity between proteins and DNA/RNA and determines whether mutations could disrupt cellular processes. Overall, the successful implementation of both DeepSEA and DeepBind methods undoubtedly illustrates the advances in non-coding mutations effect annotations.

Also, approximately 10% of disease-causing mutations are mutations within splice site sequences at the intron-exon junctions (Krawczak et al., 2007). Thus, splice-site mutations have been generally considered deleterious (Daguenet et al., 2015). Several computational tools, such as Human Splicing Finder (HSF) (Desmet et al., 2009), GeneSplicer (Pertea et al., 2001), MaxEntScan (http://genes.mit.edu/burgelab/maxent/Xmaxentscan_scoreseq.html), NNSplice (Reese et al., 1997), and MutPred Splice (Mort et al., 2014), have been developed to interpret splice-site mutations and discriminate pathogenic and tolerated ones. One of the most widely used tools, HSF (Desmet et al., 2009), contains more than 10 algorithms including position weight matrices (PWM), maximum entropy principle, and motif comparison method, to identify splicing motifs across the imputed human sequence. It evaluates the disrupted prediction of the natural discovered splice sites. MutPred Splice, more recent developed machine learning-based (random forest) prediction tool with 21 features, targets substitutions that disrupt pre-mRNA splicing (Mort et al., 2014). A survey of the *in silico* tools that predict potential consequences of splicing mutations has been carried out by Jian et al. (2014).

Furthermore, some researchers have developed tools to investigate the effects of mutations from their protein structures using the resolved or predicted protein structures, and the protein-protein interaction (PPI) information. For example, Meyer et al. (2018) developed Interactome INSIDER (INtegrated Structural Interactome and genomic Data browsER), which is a structurally resolved, multi-scale, proteome-wide human interactome allowing to explore human disease mutations functionally. This useful network enables users to analyze disease mutations from databases or from their studies to identify enrichments in protein interaction domains, residues, and atomic 3D clustering in protein interfaces.

In conclusion, when combining computational annotation tools of different purposes, researchers can thoroughly annotate the detected mutations with comprehensive genetic information and ensure having the first step toward a global interrogation of the variant effects (Figure 1B). A proper prioritization of the variants to detect the one with functional effect is therefore crucial to translate the basic research into the clinical intervention for patients' personalized medicine treatment.

### Integrating Inherited and Common Variants to Interpret Rare Genetic Variation From Exome Studies

One of the most popular ways to interpret exome data is to analyze DNMs directly. However, only ~1 to 3 *de novo* events are usually identified in exonic regions in every individual (Iossifov et al., 2014; Turner et al., 2016; Yuen et al., 2016) and there are a plethora of inherited variants also detected by trio WES that might contribute to the disease etiology. One way to gain a more systematic view of variants effects is to combine the *de novo* and the inherited variants effectively. For this purpose, He et al. (2013) developed a statistical method, later improved by Sanders et al. (2015), the Transmission and *De novo* Association Analysis (TADA), which identifies disease risk genes by combining *de novo* and transmitted SNVs and small indels, with case-control variants data from the same samples to provide a unified statistical quantification of disease association. TADA weights multiple types of variaions differently, e.g., a LoF mutation weights more than a missense mutation, which in turn weights more than a transmitted LoF mutation. By combining *de novo* and transmitted variants in its analysis, TADA (He et al., 2013; Sanders et al., 2015) assumes that candidate genes for neuropsychiatric disorders would give different types of risks to the disease. Some variants may be causative, while others may be transmitted and play roles as contributors or modifiers. Therefore, by including the information on the inherited variants, one may be able to discover pathogenic genes in cases when DNM information is insufficient. TADA has already been successfully applied in neuropsychiatric disorder studies, such as ASD (He et al., 2013; Sanders et al., 2015; Werling et al., 2018), SCZ (Takata et al., 2014; Nguyen et al., 2017), and can easily be applied to other datasets as well.

Besides assessing whether a particular variant is associated with disease by comparing the observed frequency in cases vs. controls, it is also important to consider the mutation rate of the gene carrying DNMs. Samocha et al. (2014) introduced a statistical metric measure to assess expected vs. observed DNMs by estimating the statistical likelihood of a DNM occurring spontaneously in a gene. The model first calculates the mutation rate for a gene from SNPs in non-coding regions of the genome for all possible trinucleotide to trinucleotide changes, such as AGA evolving to ATA, ACA, or AAA. Then sequence context is considered to determine separate rates for each base changing to each other base for all bases across from the coding region and the annotated conserved splice site. By applying this approach, the consequence of various types of mutation change (e.g., loss-of-function, missense, synonymous) on the corresponding amino acid coded for is determined, and the probabilities for each outcome occurring in a gene are evaluated to create a likelihood per gene for each type of DNMs. With this, Samocha et al. was able to identify 1,003 genes that are estimated to be significantly intolerant to variations that change the coding sequence of the gene (Samocha et al., 2014).

Another approach to consider mutation rate of the risk gene carrying DNMs is by incorporating the imputation-based rare variant burden test using a follow-up cohort after the DNM identification. It has been shown that the discovery of a rare variant near a common variant might be particularly informative to clarify which of the candidate gene is pathogenic (Teslovich et al., 2010; Voight et al., 2010; Momozawa et al., 2011). Thus, applying the common variant burden test imputed to the reference SNP panel on genes carrying DNMs can be an alternative approach to assess the pathogenicity of the variants. Recently, Browning et al. (Pullabhatla et al., 2018) has implemented a DNM study pipeline that includes variant discovery and burden test with imputation to reference SNP panel across coding regions of genes. In addition to discover confident candidates, it shows the SNP with genotype imputation mainly implemented for GWAS still can be a powerful supplementary annotation to the rare variant analysis.

### Network Approaches to Enhance Understanding of the Disease Functional Pathways

With various forms of large-scale genetic association studies, researchers have detected hundreds to thousands of genetic *loci* that are involved in NDP disorder risk (Gratten et al., 2014). As a consequence, many works adopting rigorous data-driven integrative network methods have been carried out to understand how all these genetic variants contribute to the disease etiology of NDP disorders (Geschwind and Konopka, 2009; Parikshak et al., 2015). The network approaches use the experimentally measured or predicted relationships between genes to link them to each other and provide an organized structural system for placing each gene in the context of its molecular framework. Networks usually model genome-wide data by displaying molecular entities, such as genes carrying DNMs from WES or protein products of the impacted genes, as nodes and the associations between nodes as network edges. Edges can be the statistical similarities between genes, such as brain-expression correlations, or physical interactions between proteins. Edges define the network connectivity and consequently define the hierarchical structures of the nodes, which can usually be organized into a relatively small group of highly interconnected modules expected to represent functional module entities. Both the inter-modular connectivity and intra-modular connectivity are used to reflect the important biological relationships: the first one reveals a higher-order organization of the network and the latter one can identify which genes are biological modulators within modules. They are often employed in the integrative analysis pipeline to identify causal functional pathways and molecular drivers of cellular and brain-wide pathology in disorders (Carter et al., 2013b; Furlong, 2013; Mitra et al., 2013).

Here, we describe two main network approaches (Figure 1C), co-expression and protein-protein interaction (PPI), to illustrate the network analysis of genes in NDP disorders (Gilman et al., 2012; O'Roak et al., 2012b; Gulsuner et al., 2013; Parikshak et al., 2013; Willsey et al., 2013). Gene expression has been widely used to investigate biological and functional relationships between human genes. The co-expression analysis was mainly designed to explore shared expression patterns in data from different experiments, tissues, or species (Stuart et al., 2003; Zhang and Horvath, 2005; Prifti et al., 2010). As results, the co-expression network utilizes the gene expression pattern correlation between genes to generate links between nodes to relate disease genes to each other (e.g., DNM genes) for the systems-level analysis, followed by the module discovery to identify topologically highly connected network modules (Cline et al., 2007; Amar et al., 2013; van Dam et al., 2017). Next, one can perform the module functional enrichment analysis to identify possible disease or brain-related pathways by using pathway databases such as the Gene Ontology (GO) (Ashburner et al., 2000), the Kyoto Encyclopedia of Genes, and Genome Elements (KEGG) (Kanehisa and Goto, 2000), with the aim to facilitate the discovery of potential therapeutic targets or biomarkers. The multiple publications in different disorders have demonstrated the effectiveness of this method in identifying disease related pathways, such ASD (Parikshak et al., 2013, 2016; Gupta et al., 2014) or SCZ (Fromer et al., 2014). For instance, Fromer et al. (2014) has applied the co-expression network approach on the set of genes hit by DNMs in patients and identified co-expression modules related to neuronal functions, including axon guidance, postsynaptic membrane, which supported the significance of the findings. Also, co-expression network analysis has also been successfully applied in cross-psychiatric-disorder studies which analyzed collective DNMs to investigate the genetic convergence among psychiatric disorders (Shohat et al., 2017; Gandal et al., 2018). For example, Gandal et al. found an astrocyte-related module significantly up-regulated in ASD, BD, and SCZ, and enriched for glial cell differentiation and fatty-acid metabolism pathways.

Another important characteristic of the biological systems is that proteins function in pairs and groups by interacting with other molecules (e.g., DNA, RNA) to regulate metabolic and signaling pathways, cellular processes, even organismal systems. As a result, PPI integrative network methods can be used to relate disease risk genes (e.g., DNM genes) to each other and their topological locations within the network modules in order to identify the potential diseases pathways. An essential organized network can be constructed by using experimentally measured or predicted associations to place each gene carrying DNM in the context of its molecular system (Geschwind and Konopka, 2009). Thus, PPI networks have emerged as a powerful resource, together with other information, to complement genetic data such as DNMs to elucidate causal molecular drivers of cellular, circuit level, and brain-wide alterations in pathology (Bergholdt et al., 2007; Lage et al., 2008; Neale et al., 2012; O'Roak et al., 2012b) (Figure 1D). For example, DAPPLE (Rossin et al., 2011) was developed to search for the significant physical connectivity among proteins encoded by disease risk genes according to PPIs reported in the literature. Neale et al. (2012) and Xu et al. (2012) have applied DAPPLE to determine whether there is an over-represented of PPIs among the genes hit by a functional *de novo* event in their results from trio WES studies in NDP disorders. The fact that different types of mutations, such as *de novo* SNVs and *de novo* CNVs, can be discovered from genome-scale studies, a network-based analysis that considers all types of mutations can be a powerful strategy for system-level understanding of the disease. To this end, NETBAG+ (Gilman et al., 2012), has been developed to consider multiple lines of mutational data from diseases, such as *de novo* SNVs, *de novo* CNVs, and SNP data from GWAS studies, and performed the PPI network-based integrative analysis to investigate the convergence of heterogeneous neuropsychiatric genetic variation on a functional system level.

In addition, some studies have combined PPIs and gene expressions to perform an integrative network analysis to better elucidate the underlining genetic basis of sets of interested genes, such as the significance of the interconnection between detected genes hits by DNMs and the overall impact of the variants in NDP disorders and have achieved successful results (Gulsuner et al., 2013; Hamdan et al., 2014; O'Roak et al., 2014; Lin et al., 2015). Supplementary Table 3 shows the characteristics and differences between different network-based methods.

## Progress and limitations in translating NGS from research to clinical implementation

Traditionally, diagnosing individuals with neuropsychiatric disorders is a very long and tedious process, and includes a large set of clinical assessments, such as dysmorphology evaluation, development monitoring, intellectual function assessment. With increasing acknowledgment of a strong genetic influence to the psychiatric disorders, especially in ASD and ID/DD, with subset of cases with an underlying genetic syndrome, clinical laboratory testing to find genomic variants in risk genes is now an important part of the diagnostic work. Genomic risk variants scanning would be considered when the clinical symptoms and test results suggest a suspected diagnose. This strategy resulted in a diagnosis rate from 5 to 50% in cases (Battaglia et al., 1999; Battaglia and Carey, 2003; Challman et al., 2003; Moog, 2005; van Karnebeek et al., 2005). However, clinicians often tussle with the diseases' phenotypic diversity and are challenged to select a proper genetic testing for complex cases.

### Current NGS Gene-Panels Commercially Available for Clinical Genetic Testing

Currently, the recommended laboratory genetic test for disorders, such as ASD and ID/DD, is the chromosomal microarray (Miller et al., 2010), targeting deleted and duplicated segments of DNA (CNVs) that only account for 5–25% of cases (Tammimies et al., 2015). In contrast, the NGS technology provides in-depth view of genomic landscape and can be used to target a selection of genes of interest by targeted gene panels, the entire coding portions of all genes by WES, or the entire genome by WGS to gain a comprehensive map of the genomic variants. Compared with the sequential testing of multiple genes historically, NGS allows the rapid sequencing of various genes simultaneously at a lower cost and is expected to improve clinical diagnosis of disorders and patients' management. NGS has proven indispensable to discover new neuropsychiatric disorder risk genes. In addition, integrative analyses of NGS data have been fruitful in illuminating the underlining genetic basis of disease etiology. It has also been commercially available as a second-tier genetic test and has demonstrated to be a powerful supplementary technique for both risk gene discovery and clinical application in disease diagnosis (Figure 1F) (Bainbridge et al., 2011; Kingsmore et al., 2011; Boycott et al., 2013).

As result, a number of NGS-based clinical testing panels have been developed and are available as NGS-based Laboratory Developed Tests (LDTs) for a set of neurodevelopmental disorders, such as Fragile X syndrome, Prader-Willi syndrome, and Angelman syndrome. These panels are useful to target a limited number of well-demonstrated causative genes involved in these disorders. However, for many psychiatric disorders with complex genetic basis, the number of genes involved is very large and the degree of confidence of the implication of these genes in disorders is in constant re-evaluation. Currently, most of available commercial genetic testing panels are for two types of complex psychiatric diseases: autism and intellectual disability. Hoang et al. (2018) has performed a comprehensive survey in the clinical sequencing panel test for ASD and found a significant heterogeneity among different laboratories with respect to the tests they offer. Their most striking finding was the number of tested genes on panels used for ASD vary from 11 to 2,562, with little overlap. Here, we performed a similar comparison on NGS-based clinical testing panel used for ID/DD and looked at the number of genes included in panels. We also found a large difference between different panels and included genes can range from 13 to 2,562 (Table 2), which is not surprised since the genetic predisposition may be different in almost every individual in these complex psychiatric disorders due to their heterogeneous nature. This shows that it is still a challenging task to reach consensus gene lists for testing panels in complex psychiatric disorders currently; and indicates that WES might be a better alternative for genetic testing when casual variants are not known since it targets all coding regions of the genome without biases in the gene pre-selection. Thus, targeted gene panels would be ideal for analyzing specific mutations or genes that have suspected associations with disease, while WES could be a better choice when one is uncertain what genes need to be tested.

**Table 2:** Clinical intellectual disability gene sequencing panels available as of January 2019.

**Laboratory company**	**# of genes included**	**Test name**
Ambry Genetics	140	Intellectual Disability (IDNext)
ApolloGen	114	X-Linked Intellectual Disability Panel
Blueprint Genetics	99	X-linked Intellectual Disability Panel
Centogene	178	X-linked Intellectual Disability Panel
CGC Genetics	89	Intellectual Disability, X-linked NGS panel
DDC Clinic	13	Overgrowth and Intellectual Disability NGS Panel
EGL Genetics	92	X-linked Intellectual Disability: Sequencing Panel
Fulgent Genetics	495	Intellectual Disability NGS Panel
Gene DX	2562	Autism/ID Xpanded Panel
GGC (Greenwood Genetic Center)	114	X-Linked Intellectual Disability (XLID) Sequencing Panel
Human Genetics Laboratory (University of Nebraska Medical Center)	117	Autism / Intellectual Disability / Multiple Anomalies Panel
Humangenetik	867	Complete Panel (#017): Intellectual Disability
MGZ (Medical Genetics Center)	115	X-Linked Intellectual Disability Panel
MNG Laboratories	894	Comprehensive Intellectual Disability/Autism + MtDNA Panel
Prevention Genetics	256	X-Linked Intellectual Disability Sequencing Panel with CNV Detection
Transgenomic	119	Autism and Intellectual Disability NGS Panel

It is worth mentioning that WGS starts to show its advantage over WES, such as providing a more uniformed coverage, able to identify more variants covering both coding, and non-coding regions of the genome (Wilfert et al., 2017). It is important to extend the interrogation to non-coding variants as these represent the majority of DNMs per genome. A relevant role in neurodevelopmental disorders of these regions such as the non-coding RNAs has already been established (Wanke et al., 2018). Although the current sequencing cost of WGS is still at least three times higher than WES per sample using NovaSeq technology as of January 2019 (Supplementary Table 4; https://genohub.com/), the implementation of WGS has the potential to ultimately replace WES both in research and clinical settings with a decreasing cost in whole genome sequencing and a rapid maturation of its analytical platforms.

### Translation of WES for Diagnostic of Neuropsychiatric Disorders From Research to Clinical Practice

As described above, an alternative strategy is needed that could take advantage of the recent advance of large-scale sequencing techniques and yield faster and higher diagnostic rates. The unbiased nature of WES can reduce the impact of disease variability on genetic testing strategies by equally weighing all genes and making assessments for all identified variants simultaneously in one clinical context (Shashi et al., 2014; Lencz and Malhotra, 2015). Hence, the true exonic phenotypic variability of genetic disorders can be assessed by WES. There are cases in the literature of pathogenic variants identified in patients that would never have been considered for a genetic testing based on their phenotypes (Fogel et al., 2014; Guerreiro et al., 2014; Lu et al., 2014). This also underlines a vital role for clinical input in bioinformatics analysis when estimating the likely contributions of new genetic variations in disorders of a particular patient. Several large studies have already demonstrated a diagnostic yield of 25–45% for clinical WES (Dixon-Salazar et al., 2012; Yang et al., 2013, 2014; Lee et al., 2014; Stark et al., 2016). Moreover, Stark et al. (2016) found that singleton WES as a first-tier screening method, outperforms the standard care in infants with suspected Mendelian disorders (57.5 vs. 13.75% diagnosis rate).

Several large diagnostic sequencing laboratories/institutions, such as Ambry Genetics Laboratory (Rossi et al., 2017), have published studies on the efficacy of diagnosis in patients with suspected genetic disorders using the exome sequencing. They have shown particularly effective diagnosis rates in patients with neuropsychiatric or neurodevelopmental diseases based on Mendelian traits, as shown in Table 3. For instance, in the field of psychiatric disorders of early onsets, such as ASD, the genetic diagnostic yield was almost doubled when WES was used in addition to chromosomal microarrays (Tammimies et al., 2015; Rossi et al., 2017). The implementation of WES in the field of rare pediatric disorders has already shown encouraging success rates, 28–40% when proband-only or trio WES were considered (Wright et al., 2018). Moreover, a pediatric neurology study obtained a higher rate of conclusive diagnoses not exceeding the economic cost supporting the usage of WES as the first-tier diagnostic test (Vissers et al., 2017). Overall, the promising results regarding time and diagnostic rates from these psychiatric studies are genuinely encouraging.

**Table 3:** Rates of diagnosis for psychiatric disorders using clinical exome sequencing.

**Study**	Rate of psychiatric diagnosis^*^	**Rate of *de novo* event**	**Global average year**	**Phenotype**
Yang et al., 2013	26% (55/213)	47% (29/62)	5–18 years (94 individuals)	neurologic disorder
			<5 years (124 individuals)	neurologic disorder and other organ-system disorder
			>18 years (28 individuals)	specific neurologic disorder
			fetus (4 individuals)	non-neurologic disorder
Yang et al., 2014	26% (455/1756)	72% (248/345)	5–18 years (845 individuals)	neurological disorder
			<5 years (900 individuals)	neurological plus other organ systems disorder
			>18 years (244 individuals)	specific neurological disorder
			fetus (11 individuals)	non-neurological disorder
Lee et al., 2014	26% (175/673)	50% (63/127)	5–18 years (266 individuals)	developmental delay
				developmental delay and other syndrome
			<5 years (254 individuals)	ataxia and related neurological disorders
				muscular dystrophy and related disorders
			>18 years (294 individuals)	cardiomyopathy and arrhythmia
				cancer predisposition
				disorder of sexual development
				Retinal disorders
Farwell et al., 2015	31% (99/324)	49% (80/163)	prenatal (2 individuals)	intellectual disability and/or developmental delay
			0–3 months (12 individuals)	brain MRI positive
			<1 years (36 individuals)	multiple congenital anomalies
			1–5 years (194 individuals)	seizures/epilepsy
			5–12 years (117 individuals)	progressive phenotype
			12–18 years (58 individuals)	ataxia
			18–40 years (45 individuals)	autism spectrum disorder
			>40 years (36 individuals)	psychiatric abnormality
Tammimies et al., 2015	3% (8/95)	38% (3/8)	average 4.5 ± 1.7 years	Asperger syndrome
				autistic disorder
				pervasive developmental disorder - not otherwise specified
Wenger et al., 2017	10% (4/40)	100% (4/4)	2–5 years (10 individuals)	neurologic abnormality
				congenital anomalies
			5–10 years (12 individuals)	metabolic abnormality
				musculoskeletal abnormality
			<2 years (9 individuals)	cancer
				gastrointestinal abnormality
			>10 years (9 individuals)	hearing loss
				vascular abnormality
Stark et al., 2016	58% (46/80)	35% (16/46)	0–6 months (37 individuals)	congenital abnormalities and dysmorphic features
				neurometabolic disorder
			6–12 months (25 individuals)	skeletal dysplasia
				eye abnormality
			12–36 months (18 individuals)	other abnormality (gastrointestinal, renal, immunological)
Rossi et al., 2017	26% (42/163)	62% (26/42)	average 9.0 ± 6.7 years	neurologic abnormality
Baldridge et al., 2017	43% (67/155)	51% (34/67)	average 6 years (3 days−33 years)	neurological abnormality
				multiple congenital anomalies
				mixed, neurological plus
				other clinical specifics
Smith et al., 2017	69% (64/96)	–	–	–
Nambot et al., 2017	15% (24/156)	50% (64/128)	average 10.5 years	congenital anomaly without intellectual disability
				neuromuscular disorders
				neurodevelopmental disorders
Vissers et al., 2017	29% (44/150)	30% (13/44)	average 5.58 years (5 months−18 years)	complex neurological disorders

* *Rate of psychiatric diagnosis refers to the percent of patients for whom were given a positive genetic diagnosis in this study; Overall diagnostic rate (all presentations) refers to the percent of patients for whom were given a positive genetic diagnosis in this and previous study; Rate of de novo event refers to the percent of events that patients carrying DNM(s) were diagnosed as positive result; Sex ratio and Global average year declaim the sex and age contribution of patient samples*.

### Challenges of NGS Application in NDP Disorders Clinical Diagnosis

The step toward the implementation of NGS technologies in large-scale clinical practice is still limited and variable across countries. The evaluation of the cost-effectiveness and management of these test remains extremely difficult (Payne et al., 2018). The application of WES in clinical practice as a diagnostic tool for neuropsychiatric disorders is challenging, overall. It is well-known that NDP disorders have strong genetic components (Gandal et al., 2016), hold a high degree of genetic and clinical heterogeneity, and present with variable expressivity and penetrance. Also, it has been demonstrated that diverse NDP disorders share genetic etiology (Cross-Disorder Group of the Psychiatric Genomics Consortium et al., 2013). Hence, using WES is a promising approach to elucidate the genetic causes of NDP disorders, however, multiple concerns remain to be addressed.

A major concern for WES, in general, is the interpretation of the results. The first challenge is how to elucidate the pathogenic effects of the identified variant(s). Multiple prediction tools and algorithms have been developed (section Interpreting Rare Genetic Variants From Exome Studies), but no gold standard interpretation guide could undoubtedly explain the causality or benignity of variants. Enormous databases of large populations' sequencing data are available, such as 1000 Genome Project (Gibbs et al., 2015) or ExAC (Lek et al., 2016), to filter out variants that are common in the population and to rank the mutation tolerance of genes. However, large datasets of multiple populations still need to be compiled. The American College of Medical Genetics and Genomics (ACMG) published guidelines that have helped to manage clinical molecular genetic cases (Richards et al., 2015; Nykamp et al., 2017; Strovel et al., 2017). In particular, recommendations on reporting incidental findings are essential in the clinical application of global genomic approaches.

Our current knowledge of NDP disorders' biological mechanisms is still limited, and the contribution of the majority of the genes in the genome to these phenotypes remains unclear. Some disease-related gene databases as ASD genes database SFARI (Abrahams et al., 2013) have introduced a scoring parameter that ranks from high confidence to hypothesized but untested, which is extremely useful to evaluate the association of a variant within these genes to the neuropsychiatric phenotype. Besides, the contributing effect of susceptibility or modifier genes remains to be systematically quantified. Therefore, advancements in knowledge moving toward these types of gene and disease potential associations will be an excellent way to help the variant classification.

WES *per se* presents some technical limitations. For example, WES has a low efficiency in detecting microsatellite expansions, which nowadays, can only be overcome using alternative techniques such as PCR or Southern blot. WES also presents a limited power to detect small CNVs and mosaic events, which require a much deeper read coverage than the regular clinical WES. Another limitation in neuropsychiatric disorders is the types of tissues studies, as brain tissues from the living individuals are not possible to obtain, the detection of brain-specific mosaic events particular to these tissues would be missed. Therefore, the complete picture of the genomic alterations of neuropsychiatric disorders will be difficult to achieve.

Elucidating the complex nature of the underlying genetics of neuropsychiatric disorders will ultimately require sophisticated mathematical models that include a large number of parameters extracted from genomic, phenotypic information, pharmacogenomics interactions, and environmental factors, among others. These complex approaches will only be reached when systematic high throughput multi-omic studies are applied to each patient, and consensus annotation terms and pipelines are used (precision medicine). Compared to difficulty in characterizing the underlying genetics of neuropsychiatric disorders, some efforts toward regulating the phenotypic terms encountered in human diseases have been made. Human Phenotype Ontology (Köhler et al., 2017) initiative and gene-associated phenotypes database as Online Mendelian Inheritance in Man® (OMIM®) are still struggling to build a standardized vocabulary of phenotypic abnormalities and its likely genetic causes. Furthermore, recently developed platforms of phenotype-genotype relationship sharing, by The Matchmaker Exchange (Philippakis et al., 2015), are already connecting worldwide clinicians and researchers with the aim to link clinical and genetic information and to identify novel genes causative of rare disease phenotypes.

Finally, the extended consequences of reaching a genetic diagnosis in neuropsychiatric disorders are especially relevant for family members and caretakers. Besides a better selection of therapeutic strategies, more accurate prognosis, appropriate support, and surveillance, risk estimation and counseling are essential for the future familial organization and reproductive planning not only for parents but also for other siblings. The application of WES requires multidisciplinary teams with a core of medical geneticist and genetic counselors who will help patients to understand the overwhelming information derived from these complex tests and ensure they make informed decisions (Paneque et al., 2017).

## Limitation of the Current Review

We acknowledge that there are many areas of WES in neuropsychiatric disorders that were not deeply addressed within this review, such as certain statistical parameters like effect size, and comparison between different statistical methods used by different tools, since they could not be easily summarized and compared, as not all studies used the same criteria. In writing this review, we relied primarily on the DNM data from denovo-db (Turner et al., 2017), which collects DNMs from large cohort studies from the past 10 years and might be missing data from some small cohort studies. In addition, the impact of DNMs in disease etiology might vary in different disorders, it exerts a large contribution for ASD and ID/DD and have smaller contributions from other disorders, such as BD and OCD, which might be because of a lack of studies or data. Therefore, how the exome case-control study might be used as a complementary strategy to the trio-based study for those disorders can be an excellent topic for a future review.

The current review is intended to serve as a broad summary, analysis, and application of neurodevelopmental and psychiatric DNVs from WES. We believe that future studies and reviews that approach genomic variants from a different angle with different NGS technologies, such as WGS or by focusing on the finer statistical details, even going so far as to scrutinize clinical data from individuals, and to link them to the variants for the interpretation, would lead to interesting and important findings.

## Conclusion and Future Directions

Despite many complications and challenges associated with NDP disorders, increasing implications of *de novo* event's contribute to the disease etiology, together with downstream functional analyses to explore the disrupted biological process by these *de novo* events, indicate the advent of the application of WES in potential treatments. Moreover, a large number of ASD drugs currently in the pipeline (Sung et al., 2014) keeps us optimistic about the future. The application of WES in clinical practice has the potential to generate an extraordinarily large dataset for multiple disorders. Obtaining large cohorts in research studies, particularly for rare neuropsychiatric diseases, is very difficult or even impossible. As a consequence, it is imperative that clinicians and researchers find a comprehensive protocol to share the genetic information and perform powerful genetic and genomic studies of diseases, always under strict data sharing protocols preserving patients' confidentiality. In the foreseeable future, we will see the development of well-established and tested systematic computational pipelines to integrate genetic and genomic data with expression, interaction and other data that will ultimately facilitate the implementation of NGS into the clinical practice.

## Author Contributions

GL designed the concept. GL and WW researched the data. GL, WW, and RC all contributed to the content discussion, writing, and editing of the manuscript.

### Conflict of Interest Statement

The authors declare that the research was conducted in the absence of any commercial or financial relationships that could be construed as a potential conflict of interest.
